# Influence of the extent of cervical lymph node dissection and lymph nodes metastases on prognosis in a cohort of dogs with oral malignant melanoma treated by surgical resection and adjuvant anti-CSPG4 electrovaccination: a retrospective study on 77 cases

**DOI:** 10.3389/fvets.2025.1616419

**Published:** 2025-07-25

**Authors:** Davide Giacobino, Matteo Olimpo, Erica Ilaria Ferraris, Greta Martinelli, Lorella Maniscalco, Mariateresa Camerino, Federica Riccardo, Federica Cavallo, Lidia Tarone, Marzia Cino, Alfredo Dentini, Selina Iussich, Elena Lardone, Luca Manassero, Raffaella De Maria, Paolo Buracco, Emanuela Morello

**Affiliations:** ^1^Department of Veterinary Sciences, University of Torino, Torino, Italy; ^2^Animal Oncology and Imaging Center AG, Hünenberg, Switzerland; ^3^Department of Molecular Biotechnology and Health Sciences, Molecular Biotechnology Center, University of Torino, Torino, Italy; ^4^Department of Medical Veterinary Science, University of Parma, Parma, Italy; ^5^Clinica Veterinaria Tyrus, Terni, Italy; ^6^MyLav La Vallonea, Veterinary Analysis Laboratory srl, Alessano, Italy

**Keywords:** lymph node, oral malignant melanoma, dog, CSPG4, lymph node dissection

## Abstract

The most appropriate approach to regional/sentinel lymph nodes (LN) for staging canine oral malignant melanoma (OMM) is still controversial. This study aims to retrospectively evaluate the prognostic impact of neck dissection modality and LN metastasis in a homogeneous cohort of dogs treated by surgery and adjuvant anti-CSPG4 electrovaccination. Seventy-seven dogs were enrolled and divided into two groups based on the presence (Group A, 24 dogs) or absence (Group B, 53 dogs) of histologically confirmed LN metastasis at the time of surgery. The overall LN metastatic rate was 31%; metastasis was found mostly in the mandibular lymph center (83%). Median survival time (MST) and disease-free interval (DFI) in Group A were 406 and 134 days, respectively. Although shorter, these values were not significantly different from MST and DFI in Group B (534 and 219 days, respectively; *p* = 0.16 and *p* = 0.11). Stratifying the cases based on the type of lymphadenectomy performed, no statistical differences were observed between Groups 1 (ipsilateral lymphadenectomy) and 2 (bilateral lymphadenectomy) regarding both MST and DFI. Similarly, no significant differences in MST and DFI were observed among subgroups based on ipsilateral (Group 4) and bilateral (Group 6) removal versus ipsilateral (Group 3) and bilateral (Group 5) non-removal of even the medial retropharyngeal LN. No association was found between LN metastasis and recurrence or distant metastasis. Finally, no association was found between lymphadenectomy pattern and progressive disease. The results recorded in this study, i.e., that ipsilateral mandibular lymphadenectomy may be a reasonable surgical option in OMM, apply for this cohort of dogs only, and the translation of this principle to canine OMMs differently treated needs further investigations. Additionally, further efforts should be addressed to studies on sentinel LN identification for canine OMM staging.

## Introduction

Canine oral malignant melanoma (OMM) is an aggressive tumor characterized by rapid growth and high local invasiveness; the reported metastatic rate, from presentation to post-treatment follow-up, is from 30.3 to 74.0% in regional lymph nodes (LN) and from 14 to 92% in lungs and other distant sites (1–6).

The biological behavior of canine OMM can be predicted evaluating the tumor size and volume, clinical stage, presence/absence of bone invasion, and histological and immunohistochemical factors, such as the degree of Ki67 expression, the mitotic count (MC), nuclear atypia (1, 2, 4, 7–11, 58, 59).

The current standard of care for local control of canine OMM consists of wide surgical excision, including 1.5–2 cm of macroscopically normal tissue and adjacent bone if it is part of the excision margin (3, 5, 8, 12, 13). In cases of incomplete excision, non-resectable tumors or if owners refuse surgery, radiotherapy should be considered (14, 15). Electrochemotherapy may be proposed as an alternative, although its efficacy is limited in presence of bone involvement (16, 17).

Despite the achievement of local tumor control, most dogs with OMM die or are euthanized because of distant metastases. Adjuvant chemotherapy, primarily based on carboplatin administration, has not been proved to significantly improve survival, particularly in stage III and IV OMMs, where the median survival time remains less than 1 year (12, 13, 18–22). Metronomic therapy may offer some palliative benefits in dogs with oral cancers, including MM, although further validation is needed (22, 23).

Given the immunogenic features of OMM, immunotherapy has emerged as a promising adjuvant treatment (60). Melanoma-associated antigens have been used to generate vaccines able of evoking an immune response against canine OMM, specifically tyrosinase (24–28, 57) and chondroitin sulfate proteoglycan 4 (CSPG4) (2, 3, 29–31).

Despite a *consensus* has been reached regarding OMM local control, modalities of neck dissection for clinical staging and their impact on prognosis remain an area of ongoing research and debate (3, 5, 12, 32).

It is well-established that a reliable assessment of the nodal metastatic status should be based on histopathology of the excised nodes, even when they appear clinically and/or cytologically normal (33–37). Given that and considering the propensity of OMM to unpredictably metastasize to homolateral and contralateral cervical nodes, an aggressive surgical approach to those nodes seem to be warranted to accurately assess nodal involvement and therefore correctly define the N parameter of the TNM clinical tumor staging. Consequently, elective neck dissection is often performed to ensure removal of all potentially affected nodes (34, 38). Alternatively, to balance the need for accurate staging with the objective of minimizing the surgical dose and the potential complications associated with an aggressive neck dissection, sentinel LN identification via Computed Tomography (CT) indirect lymphangiography (CTL) alone or in association with intraoperative blue dye or near infrared fluorescence imaging (NIRF) with indocyanine green (ICG), lymphoscintigraphy or contrast-assisted ultrasound (CEUS) has been also investigated (39–44), and some promising results have been reported.

Elective neck dissection and bilateral nodal extirpation and sentinel lymph node biopsy (SLNB) have been proposed, but there are no data if any of those is superior or yields a prognostic benefit for the patients (45, 46).

In this retrospective study, the authors aim to assess the prognostic impact of LN metastases in dogs with OMM treated with surgical excision of the primary tumor and adjuvant anti-CSPG4 electrovaccination. Specifically, the study investigates how different types of lymphadenectomies, ranging from a more selective to a more extensive LN-neck dissection, affect survival time (ST) and disease-free interval (DFI). In addition, the incidence and anatomical distribution of metastatic LNs in the cervical region are analyzed to explore potential associations with clinical outcomes. By focusing on a homogeneous cohort of patients receiving the same therapeutic protocol, the study aims to minimize confounding factors and to better identify the prognostic relevance of nodal involvement and of the extent of neck dissection.

## Materials and methods

### Patients’ selection and data collection

Client-owned dogs affected by OMM, which were presented at the Veterinary Teaching Hospital of Grugliasco (Turin, Italy) from December 1st, 2014, to December 31st, 2023, were retrospectively considered for this study. Dogs were referred for either surgical treatment or adjuvant anti-CSPG4 DNA electrovaccination or both. Dogs were treated according to the Good Clinical Practice guidelines for animal clinical studies; a specific written consent form was signed by the owners for animals’ enrollment in the electrovaccination study, and for anesthetic, diagnostic and surgical procedures. Both the Ethics Committee of the University of Turin and the Italian Ministry of Health had approved the immunotherapy trials (0004230–20/02/2018-DGSAF-MDS-P and 0015537–28/06/2017-DGSAF-MDS-P).

Inclusion criteria for this study were: (a) complete staging, consisting of either a CT scan or three-view thoracic radiographs and abdominal ultrasound, with no evidence of distant metastases at presentation, (b) no concurrent life-threatening disease (mild to severe renal, hepatic or cardiac diseases or other simultaneous tumors), (c) surgical excision of the primary OMM concurrent with regional lymphadenectomy (ipsilateral or bilateral mandibular and/or medial retropharyngeal LNs), (d) definitive histological diagnosis of OMM, (e) immunohistochemical CSPG4 positivity, (f) histology of the cervical excised LNs and their classification as metastatic or not metastatic, (g) adjuvant anti-CSPG4 electrovaccination. Each dog included in the study had a minimum follow up of 1 year.

For each dog, the collected data included age, gender, weight, breed, tumor size and localization, clinical and at imaging enlargement of cervical LNs (mandibular and medial retropharyngeal), type of surgery performed (wide surgical excision of OMM, including mandibulectomy and maxillectomy or marginal resection) and pattern of regional lymphadenectomy (ipsilateral or bilateral mandibular +/− medial retropharyngeal nodes), complete TNM stage after LN histology and adjuvant treatment performed (anti-CSPG4 DNA electrovaccination; second surgery and/or radiotherapy or electrochemotherapy at local recurrence occurrence; and metronomic therapy at disease’s progression). Only dogs bearing an OMM with a CSPG4 score ≥ 3/8 were suitable for vaccination. Briefly, a modified semi-quantitative scoring system was adopted to evaluate membrane staining in 10 randomly selected high-power fields (400x) within the tumor. A score representing the estimated proportion of positively stained tumor cells was assigned as follows: 0 (none); 1 (<1/100); 2 (1/100–1/10); 3 (1/10–1/3); 4 (1/3–2/3); and 5 (>2/3). An intensity score was also assigned to represent the estimated average staining intensity of positive tumor cells (0, none; 1, weak; 2, intermediate; 3, strong). The proportion and intensity scores were then summed to obtain a total score ranging from 0 to 8. Samples with a total score greater than or equal to 3 were considered positive (47).

For all the canine OMM samples the following histological and immunohistochemical data were recorded: excision margin status (surgical margins were considered complete if the narrowest histologic margin was >2 mm), LN status (metastatic or not metastatic), presence of bone invasion, MC (cut-off of 4/10 high power field - hpf), score of Ki67 expression (using polyclonal Ki67 antibody A-047; DAKO; the Ki67 labeling index was determined by counting the number of positively labeled neoplastic cell nuclei within the area of a 1 mm2 optical grid reticle at 400×. Five grid areas within the highest labeling were counted and averaged to determine the Ki67 labeling index. Areas under regions of ulceration were avoided. Cut-off of 19.5) (2, 9, 48, 61) and CSPG4 immunohistochemical score (47, 62).

### Staging, study design and treatment

Before surgery, all dogs underwent a thorough physical examination, blood exam (complete blood count and serum biochemistry) and urinalysis. For both clinical staging and surgical planning, a pre-operative total body CT scan was accomplished; alternatively, according to the owners’ decision, skull radiographs, three views chest radiographs and abdominal ultrasound were carried out.

All the dogs of this cohort underwent a surgical excision of the OMM and regional lymphadenectomy, then dogs were adjuvantly treated with the anti-CSPG4 DNA electrovaccination. Under brief general anesthesia, dogs were vaccinated with plasmids coding for the CSPG4 antigen, as already described (2, 3, 30, 31). The vaccination was started after surgery and repeated after 2 weeks and then monthly for a minimum of 6 and a maximum of 24 immunizations. According to the vaccination protocol, the dogs were re-evaluated monthly for the initial 6 months, and every 3 months thereafter. Examination consisted of clinical assessment, blood work, and CT scans of the head, neck, and thorax. Abdominal ultrasound examinations were conducted every 3 months (29).

Initially, dogs were stratified based on the presence (A) or absence (B) of regional LN metastasis at presentation. Dogs were then categorized according to the type of LN extirpation performed. Group 1 included dogs that underwent ipsilateral LN dissection, while Group 2 included those that underwent bilateral LN dissection. Group 1 was further subdivided based on the lymph centers removed: only the mandibular LNs (Group 3) or both the mandibular and medial retropharyngeal LNs (Group 4). The same subdivision was applied to Group 2, resulting in Groups 5 and 6, respectively (Table 1). Given the low rate of metastasis in canine oral cancers, parotid LN excision was not considered in this study.

**Table 1:** Stratification of the study population.

Overall population (77)	
Presence Lymph Node Metastases Group A	Absence Lymph Node Metastases Group B
24	53

Overall population (77)	
Ipsilateral Lymphadenectomy Group 1	Bilateral Lymphadenectomy Group 2
45	32

Mandibular Group 3	Mandibular and Retropharyngeal Medial Group 4	Mandibular Group 5	Mandibular and Retropharyngeal Medial Group 6
34	11	12	20

### Statistical analysis

The analyses were carried out using GraphPad Prism (version 10.3.1 for Windows, GraphPad Software, San Diego, California^1^), with statistical significance set at a *p* < 0.05.

The data were summarized using descriptive statistics and were indicated as median and range. Distribution was checked graphically using the Shapiro–Wilk Test; Mann–Whitney and Kruskal-Wallis tests were used to assess statistical differences among different groups regarding age, weight, stages, MC, Ki67, CSPG4 expression and clinical tumor stage. The disease-free interval (DFI) and the median survival time (MST) were estimated using the Kaplan–Meier method, and differences in DFI and MST among treatment groups were assessed with the log-rank test. A power analysis for the log-rank test was performed using the online MedCalc software^2^ (see Appendix material). The DFI of dogs was calculated from the day of surgery to the first tumor recurrence or metastasis while the ST was defined as the period from the day of surgery to the patient’s death. Dogs which died from OMM unrelated causes, those lost to follow-up and those still alive at the end of the study were censored. Finally, Fisher’s exact test was used to test a potential association between the different patterns of neck dissection and the probability of local recurrence and/or metastasis and to assess the relationships between LN metastasis and primary tumor size and location within the oral cavity.

## Results

### Signalment

Seventy-seven client-owned dogs bearing an OMM entirely fulfilling the inclusion criteria were enrolled. Of these dogs, 42/77 (54.5%) were males (27 neutered and 15 intact) and 35/77 (45.5%) were females (33 spayed and 2 intact). The median age at presentation was 11 years (range, 7–15) and the median weight was 22 kg (range, 3–45 kg) (Table 2).

**Table 2:** Clinical characteristics of the dogs, and histological and immunohistochemical parameters of OMM enrolled in the study.

		Overall population (77)	Presence Lymph Node Metastases Group A (24)	Absence Lymph Node Metastases Group B (53)
Age (Years)	Median	11	11	11
	Range	7–15	8–14	7–14
Weight (kg)	Median	22	22	24
	Range	3–45	4–45	3–44
Sex (%)	Female Intact	2 (2.6%)	0 (0%)	2 (3.8%)
	Spayed	33 (42.9%)	10 (41.7%)	23 (43.4%)
	Male Intact	27 (35.0%)	8 (33.3%)	19 (35.8%)
	Neutered	15 (19.5%)	6 (25.0%)	9 (17.0%)
Tumor location (%)	Mandible	25 (32.5%)	8 (33.3%)	17 (32.0%)
	Maxilla	17 (22.1%)	4 (16.7%)	13 (24.5%)
	Cheek	12 (15.6%)	4 (16.7%)	8 (15.1%)
	Lip	16 (20.7%)	6 (25.0%)	10 (18.9%)
	Palate	5 (6.5%)	2 (8.3%)	3 (5.7%)
	Tonsil	2 (2.6%)	0 (0%)	2 (3.8%)
Tumor Size (%)	T1	20 (26%)	7 (29.2%)	13 (24.5%)
	T2	41 (53%)	11 (45.8%)	30 (56.5%)
	T3	16 (21%)	6 (25.0%)	10 (18.9%)
Margins	Tumor-free	59 (76.6.%)	20 (83.3%)	39 (73.6%)
	Infiltrated	15 (19.5%)	3 (12.5%)	12 (22.6%)
	Unknown	3 (3.9%)	1 (4.2%)	2 (3.8%)
Bone invasion	Presence	17 (22.1%)	5 (20.8%)	12 (22.6%)
	Absence	58 (75.3%)	19 (79.2%)	39 (73.6%)
	Unknown	2 (2.6%)	0 (0.0%)	2 (3.8%)
Pigmentation	Melanotic	25 (32.5%)	8 (33.3%)	17 (32.1%)
	Amelanotic	36 (46.7%)	10 (41.7%)	26 (49.0%)
	Partial Melanotic	11 (14.3%)	4 (16.7%)	7 (13.2%)
	Unknown	5 (6.5%)	2 (8.3%)	3 (5.7%)
Mitotic Count	> 4/10 hpf	67 (87.0%)	23 (95.8%)	44 (83.0%)
	< 4/10 hpf	6 (7.8%)	0 (0.0%)	6 (11.3%)
	Unknown	4 (5.2%)	1 (4.2%)	3 (5.7%)
Ki67	≥19.5	54 (70.1%)	19 (79.2%)	35 (66.0%)
	<19.5	21 (27.3%)	5 (20.8%)	16 (30.2%)
	Unknown	2 (2.6%)	0 (0.0%)	2 (3.8%)

Thirty-one percent (24/77) of this canine population was represented by crossbreed dogs, while the most represented pure breed was Golden Retrievers (10 dogs). The remaining breeds are listed in Table 3.

**Table 3:** Distribution of breeds in this cohort of dogs.

Breed	Number of dogs for each breed
Crossbreed	24
Golden Retriever	10
Labrador Retriever	6
Pinscher, Cocker English Spaniel	4
German Shepherd, English Bulldog	3
English Setter; Pekingese, Jack Russel, Miniature Schnauzer	2
Akita-Inu, Alaskan Malamute, American Staffordshire Terrier, Poodle, Dachshund, Beagle, Dogue de Bordeaux, Malinois, Pug, Hovawart, Australian Shepherd, Rhodesian, Rottweiler, Shi-tzu, Yorkshire, Shar-pei	1

### Clinical and histological characteristics

Overall, 25/77 (32.5%) dogs had an OMM on the gingiva of the lower arcade, 17/77 (22.1%) dogs on the gum of the upper arcade, and 16/77 (20.7%) dogs on the lips. Additionally, 12/77 (15.6%) dogs had an OMM at the level of the cheek, 5/77 (6.5%) dogs on the palatal mucosa, and 2/77 (2.6%) dogs in the tonsil (Table 2).

Mandibulectomy and maxillectomy were performed in 23/77 (30.0%) and 14/77 (18.0%) dogs, respectively. Twenty-seven (27/77, 35.0%) dogs underwent a wide excision (11, 13, 3 OMMs of the cheek, lips, and palatal mucosa, respectively). A marginal excision was performed by the referring veterinarian in 13/77 (17.0%) dogs, followed by a revision surgery in 5 dogs (performed within a range of 32–74 days) and by radiotherapy in 2 further dogs (started within 2 weeks after the marginal excision). In the remaining 6/13 cases, surgical revision was not performed due to the absence of macroscopic residual disease at clinical examination and staging.

Histological status of surgical margins was considered clear in 59/77 (76.6%) cases, infiltrated in 15/77 (19.5%) cases and unknown in the remaining 3/77 (3.9%) cases. Local bone invasion was detected on CT scan and histology in 17/77 (22.1%) dogs. Regarding histological and immunohistochemical prognostic factors, MC was ≥4/10 hpf in 67/77 (87.0%) OMMs, <4/10 hpf in 6/77 (7.8%) OMMs and unknown in 4/77 (5.2%) samples. The Ki67 expression was ≥19.5 in 54/77 (70.0%) OMMs, <19.5 in 21/77 (27.4%) OMMs and not available in 2/77 (2.6%) cases. The CSPG4 expression was ≥ 3 in all the OMM. Twenty-five/77 (32.5%) OMMs were melanotic, 36/77 (46.7%) OMMs were classified as amelanotic, 11/77 (14.3%) as partially melanotic, and in 5/77 (6.5%) cases the grade of pigmentation was not reported (Table 2).

A regional lymphadenectomy was performed in all dogs. Ipsilateral LNs were removed in 45 out of 77 (58.4%) dogs (Group 1) while a bilateral lymphadenectomy was carried out in the remaining 32/77 (41.6%) dogs (Group 2). In the first group, 34/45 (75.6%) dogs underwent only mandibular lymphadenectomy (Group 3) while in 11/45 (24.4%) also the medial retropharyngeal LN was extirpated (Group 4). Considering dogs that underwent bilateral LN excision (Group 2), lymphadenectomy of the mandibular LNs only was performed in 12/32 (37.5%) dogs (Group 5) while both mandibular and medial retropharyngeal LNs extirpation was done in 20/32 (62.5%) dogs (Group 6) (Table 1).

Regional LNs appeared enlarged on both clinical evaluation and CT in 24/77 cases (31.2%). Of these, only 12 (50.0%) were truly metastatic while the other 12 enlarged LNs were histologically normal. Considering the metastatic LNs, the other 12 were normal on both clinical and imaging evaluation. Indirect CTL for sentinel LN was performed only in 8 dogs that underwent a bilateral lymphadenectomy (Group 6). In 7 dogs, CTL identified the ipsilateral mandibular LN as the sentinel node. Among these, 5 LNs were histologically normal without any evidence of metastasis; in the remaining 2 dogs, CTL correctly identified the metastatic LNs. In one case, CTL identified the ipsilateral medial retropharyngeal LN as the sentinel node, but histology revealed metastasis in the contralateral mandibular LN.

Based on postoperative histology, 24 out of 77 (31.2%, Group A) dogs displayed metastatic LNs. The regional LN metastatic rate was 33.3% (15/45) in Group 1 and 28.1% (9/32) in Group 2. When analyzing metastatic involvement in the subgroups, 11 dogs (32.3%) in Group 3 exhibited mandibular LNs metastasis. In Group 4, metastases were observed in 1 dog at a mandibular LN, in 1 dog at a medial retropharyngeal LN, and in 2 dogs at both the mandibular and medial retropharyngeal LNs. In Group 5, 1 dog presented metastasis at an ipsilateral mandibular LN while another dog had bilateral mandibular LNs involvement. In Group 6, metastatic spread was identified in 4 dogs at the ipsilateral mandibular LNs, in 1 dog at the contralateral mandibular LN, in 1 dog at both bilateral mandibular LNs, and in 1 dog metastases were observed in all the LNs excised (after bilateral mandibular and medial retropharyngeal LN excision) (Table 4).

**Table 4:** Distribution of metastasis in cervical LNs.

		Ipsilateral Lymphadenectomy Group 1 (45)		Bilateral Lymphadenectomy Group 2 (32)	
		Mandibular Group 3 (34)	Mandibular and medial retropharyngeal Group 4 (11)	Mandibular Group 5 (12)	Mandibular and medial retropharyngeal Group 6 (20)
Presence Lymph Node Metastases Group A (24)	Mandibular	11	1	1 (i) 1 (b)	4 (i) 1 (c) 1 (b)
	Medial Retropharyngeal	0	1	0	0
	Mandibular and Medial Retropharyngeal	0	2	0	1 (b)

i = ipsilateral, c = contralateral, b = bilateral.

Final tumor staging was as follows:13 dogs were classified as stage I (17.0%), 30 as stage II (39.0%) and 34 as stage III (44.0%) (Table 5).

**Table 5:** Clinical staging, according to TNM classification system (55), of the dogs enrolled in the study.

		**Clinical Stage (%)**		
		Stage I	Stage II	Stage III
Overall population (77)	Presence Lymph Node Metastases Group A (24)	0 (0.0%)	0 (0.0%)	24 (100.0%)
	Absence Lymph Node Metastases Group B (53)	13 (25.5%)	30 (56.6%)	10 (18.9%)
Ipsilateral lymphadenectomy Group 1 (45)	Mandibular Group 3 (34)	2 (6.0%)	16 (47.0%)	16 (47.0%)
	Mandibular and Medial Retropharyngeal Group 4 (11)	1 (9.1%)	4 (36.4%)	6 (54.5%)
Bilateral lymphadenectomy Group 2 (32)	Mandibular Group 5 (12)	5 (41.7%)	5 (41.7%)	2 (16.6%)
	Mandibular and Medial Retropharyngeal Group 6 (20)	5 (25.0%)	5 (25.0%)	10 (50.0%)

All dogs received adjuvant anti-CSPG4 DNA vaccination, with a median of 7 vaccinations administered (range, 4–26). In case of progressive disease (lung and/or LN metastasis and/or local recurrence), 18 dogs (23.4%) also received metronomic chemotherapy consisting of a combination of piroxicam, cyclophosphamide and thalidomide.

No statistical differences were observed between the groups when considering MC, Ki67, CSPG4, bone invasion, pigmentation, clinical stage and adjuvant treatments.

### Follow up and statistical data

At the end of the study, 14/77 dogs (18.2%) were still alive (range, 367–2,167 days), while 61/77 (79.2%) had died (range, 171–2,252 days), 43 (68.3%) of which for OMM-related causes (range, 171–1,063 days), while 2/77 (2.6%) were lost to follow-up (512 and 962 days, DFI of 241 days for the latter). Sixty dogs out of 77 (78%) developed progressive disease, with 10 dogs (16.7%) experiencing a local recurrence only, 26 (43.3%) distant metastases only, 2 (3.3%) LN metastasis only, while 22 dogs (36.7%) displayed a combination of at least two among local recurrence, regional and distant metastases. Data are summarized in Table 6.

**Table 6:** Follow up of the dogs enrolled in the study divided by groups.

		Local recurrence	Distant metastasis	Lymph node metastasis
Overall population (77)	Alive (14)	1 (1)	3 (3)	0 (0)
	OMM-related death (43)	4 (24)	17 (35)	2 (9)
	Unrelated death (18)	5 (7)	5 (7)	0 (2)
	Lost to Follow-up (2)	0 (0)	1 (1)	0 (0)
Presence lymph node metastases Group A (24)	Alive (3)	1 (1)	0 (0)	0 (0)
	OMM-related death (15)	0 (7)	7 (13)	0 (3)
	Unrelated death (6)	1 (3)	1 (3)	0 (2)
	Lost to Follow-up (0)	0 (0)	0 (0)	0 (0)
Absence lymph node metastases Group B (53)	Alive (11)	0 (0)	3 (3)	0 (0)
	OMM-related death (28)	4 (17)	10 (22)	1 (6)
	Unrelated death (12)	4 (4)	4 (4)	0 (0)
	Lost to Follow-up (2)	0 (0)	1 (1)	0 (0)
Ipsilateral lymphadenectomy Group 1 (45)	Alive (7)	0 (0)	3 (3)	0 (0)
	OMM-related death (24)	3 (13)	10 (18)	1 (3)
	Unrelated death (12)	4 (5)	1 (3)	0 (1)
	Lost to Follow-up (2)	0 (0)	1 (1)	0 (0)
Bilateral lymphadenectomy Group 2 (32)	Alive (7)	1 (1)	0 (0)	0 (0)
	OMM-related death (19)	1 (11)	7 (17)	1 (6)
	Unrelated death (6)	1 (2)	4 (4)	0 (1)
	Lost to Follow-up (0)	0 (0)	0 (0)	0 (0)

The total number of dogs involved (with a combination of at least two of local recurrence, regional, and distant metastases) is provided in brackets.

Among the 32/77 dogs (41.5%), which developed local recurrence, this was further managed with radiotherapy, electrochemotherapy and surgery in 3 (3.9%), 5 (6.5%), and 18 (23.4%) dogs, respectively. Electrochemotherapy, following intravenous bleomycin infusion, was applied in an attempt to reduce the size of local recurrence. Additionally, LNs extirpation were performed in 6 dogs (7.8%) to treat the development of new LN metastases. Specifically, 3 dogs had initially undergone bilateral mandibular and medial retropharyngeal LN dissection (Group 6), 1 had received ipsilateral mandibular lymphadenectomy (Group 3), and in 2 dogs, bilateral mandibular LNs had been excised (Group 5).

**Figure 1:**
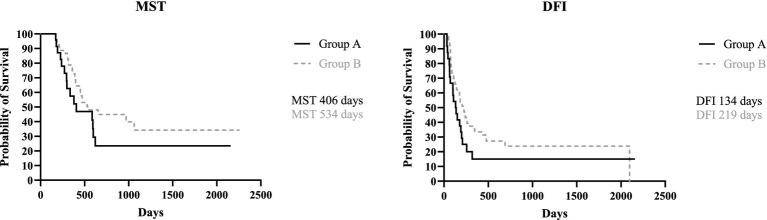
Kaplan Meyer analysis of MST (*p* = 0.16) and DFI (*p* = 0.11) of Group A and B.

When dividing the study population based on presence/absence of LN metastases, the MST for group A (metastatic) and group B (not metastatic) was, respectively, 406 days (range, 171–2,157 days) and 534 days (range, 171–2,252 days), with no statistical difference between the two groups (*p* = 0.16). The DFI for group A and B was 134 days (range, 34–2,157 days) and 219 days (range, 36–2098 days), respectively; again, no statistical difference was found (*p* = 0.11) (Figure 1).

**Figure 2:**
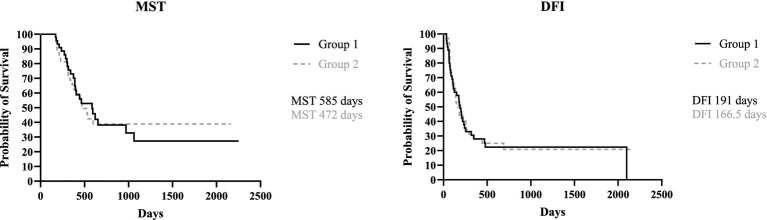
Kaplan Meyer analysis of MST (*p* = 0.98) and DFI (*p* = 0.84) of Group 1 and 2.

When dividing the canine population based on the type of lymphadenectomy performed, MST for Group 1 (ipsilateral excision) was 585 days (171–2,252 days) while MST for Group 2 (bilateral lymphadenectomy) was 472 days (range, 171–2,157 days); DFI for Group 1 was 191 days (range, 34–2098 days) and 166.5 days (range, 36–2,157 days) for Group 2, with no statistical difference found for both MST and DFI between the two groups (*p* = 0.98 and *p* = 0.84, respectively) (Figure 2).

**Figure 3:**
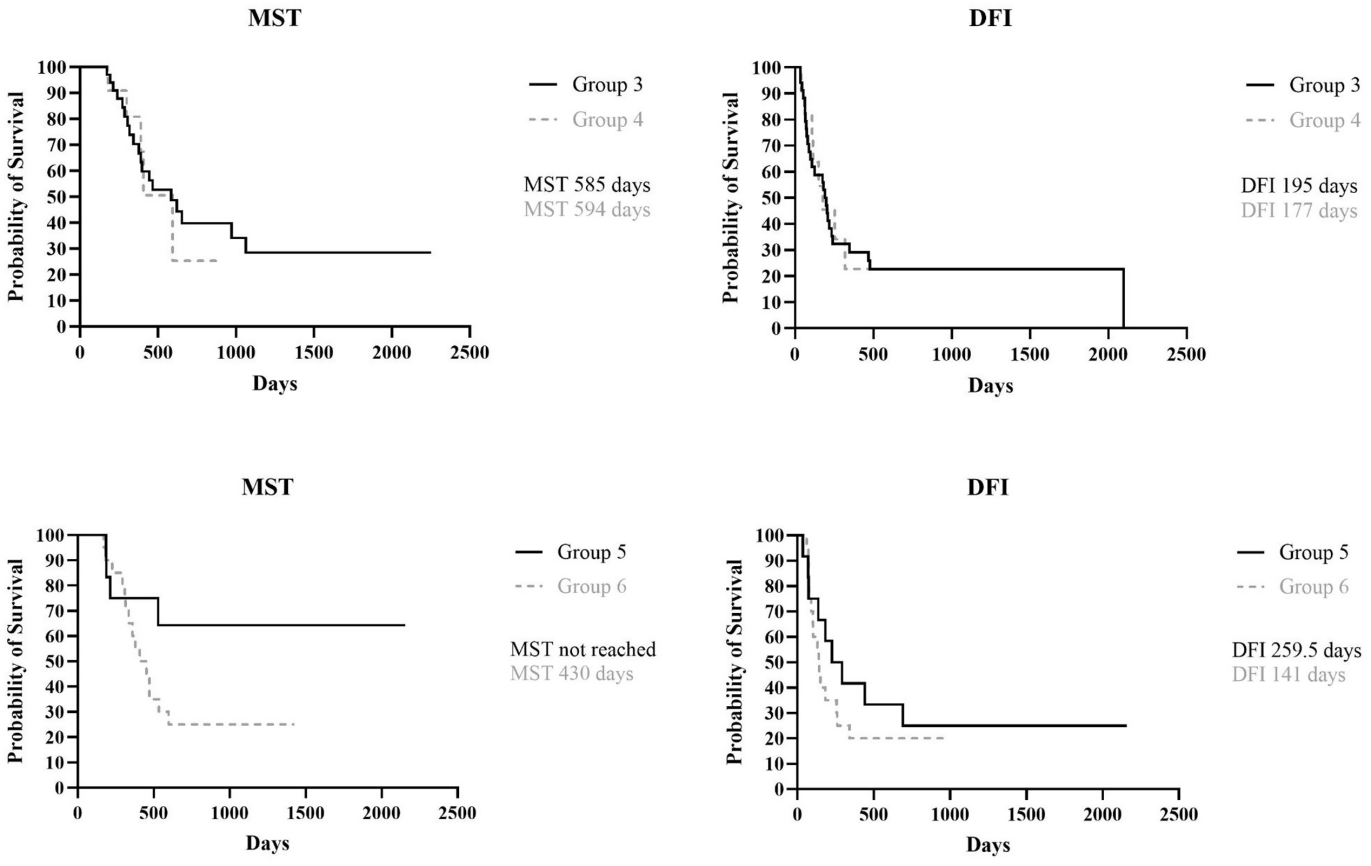
Kaplan Meyer analysis of MST (*p* = 0.86) and DFI (*p* = 0.94) of Group 3 and 4, and MST (*p* = 0.06) and DFI (*p* = 0.43) of Group 5 and 6.

The MST and DFI were then assessed in the subgroups categorized by whether or not the medial retropharyngeal LN had been surgically removed. The MST for Group 3 (ipsilateral mandibular lymphadenectomy) and Group 4 (ipsilateral mandibular + medial retropharyngeal lymphadenectomy) was, respectively, 585 days (range, 171–2,252 days) and 594 days (range, 179–920 days), while the DFI for Group 3 was 195 days (range, 34–2098 days) and 177 days (range, 39–920 days) for Group 4, with no statistically significant difference found between the two groups (*p* = 0.86 and *p* = 0.94). When Group 5 (bilateral mandibular lymphadenectomy) and Group 6 (bilateral mandibular + retropharyngeal lymphadenectomy) were compared, MST of Group 5 was not reached (range, 187–2,157 days) and, even if longer, there was no statistical difference with MST of Group 6 that was 430 days (range, 171–1,424 days) (*p* = 0.06). Similarly, the DFI of Group 5 was 259.5 days (range, 36–2,157 days), longer than that of Group 6 that was 141 days (range, 61–990 days), but no significant difference was found between the two groups (*p* = 0.43) (Figure 3).

Furthermore, when Group 3, in which fewer LNs were removed, was compared with Group 6, in which both mandibular and medial retropharyngeal LNs were bilaterally removed, no statistical differences were found for both MST and DFI (*p* = 0.34 and *p* = 0.76, respectively).

The survival rates at 6, 12, 18 and 24 months for each group are reported in Table 7.

**Table 7:** Percentages of survival in each group.

Groups	Survival rates			
	6 months	12 months	18 months	24 months
Presence lymph node metastases Group A (24)	91%	55%	42%	21%
Absence lymph node metastases Group B (53)	98%	77%	41%	32%
Ipsilateral lymphadenectomy Group 1 (45)	95%	72%	45%	29%
Bilateral lymphadenectomy Group 2 (32)	97%	69%	40%	27%

The overall MST for the entire canine population was 529 days (range, 171–2,252 days) while the overall DFI was 183 days (range, 34–2,157 days).

No statistical association was found between LNs metastasis at the time of surgery and the primary tumor dimension (*p* = 0.67) or its location within the oral cavity (*p* = 0.91).

Additionally, no association was found between the extent of lymphadenectomy performed and the disease progression (*p* = 0.99), the occurrence of distant metastases (*p* = 0.48), the incidence of local recurrence (*p* = 0.81), and the development of new regional LN metastasis (*p* = 0.18). Finally, no statistical association was observed between the presence of LN metastasis at diagnosis and the occurrence of recurrence (*p* = 0.62) or distant metastases (*p* = 0.46).

## Discussion

A *consensus* has been reached on the need for a multimodal approach to canine OMM; however the appropriate extent of surgical dose for staging the regional/sentinel LNs, as well as the potential therapeutic effect and prognostic impact of elective neck dissection on the disease progression, remain still unclear (5, 32). To fill this gap of knowledge, the present study was conducted to assess the impact of LN metastases on the long-term survival in a specific cohort of dogs with OMM. Furthermore, we aimed at evaluating if elective neck dissection and bilateral nodal extirpation (45) offers a prognostic benefit compared to a targeted – and therefore less invasive – approach to the regional/sentinel LNs.

In this study, the prevalence of histologically confirmed LN metastasis at presentation was 31.2%, consistent with previous literature (1, 4, 38). The mandibular lymph center exhibited the highest incidence of metastasis (20/24 cases, 83.0%), differing from earlier reports in which metastasis was more evenly distributed also to the medial retropharyngeal LN (34, 36).

The histological metastatic status of the excised nodes was not associated with primary tumor size or location, nor with nodal clinical size, thus corroborating the unreliability of LN clinical size to predict their status.

This result once again confirms that histopathology should be considered the most reliable method to assess nodal metastases (37, 38, 41, 43). In this study 12 enlarged LNs were histologically reactive, while 12 metastatic LNs appeared clinically normal on clinical and CT examination, as already reported in other studies (37). However, no cytology was performed on these LNs preoperatively, as their removal was already planned as part of the protocol; so no comparison with post-excisional histology was conducted in this study.

The risk of distant metastases or local recurrence was not associated with the presence of LN metastasis at the time of surgery, indicating that factors other than the nodal status may play a more critical role in disease progression. Nevertheless, it should be noted that the MST of dogs with metastatic LNs was lower than that of dogs without LN metastasis at the time of surgery although this difference was not statistically significant (*p* = 0.16); a similar trend was observed for DFI. Despite this, the clinically relevant difference in survival between the 2 groups supports the potential importance of lymphadenectomy in the management of dogs with OMM.

The most effective pattern of LN extirpation was assessed by stratifying our canine OMMs population based on the type of lymphadenectomy performed. However, the comparison among these groups did not reveal any significant differences in terms of MST and DFI. Furthermore, when comparing the group of dogs with the fewest LNs removed (ipsilateral mandibular only) with the group with the highest number of LNs excised (bilateral mandibular and medial retropharyngeal), no statistical differences were observed in terms of both MST and DFI. No significant association was found between the type of lymphadenectomy performed and either the risk of local recurrence, distant metastases, or tumor dissemination to other LNs. The lack of statistical significance here may be due to the limited sample size. However, these findings align with the “marker hypothesis” proposed for human melanoma, which suggests that LN metastases reflect a biologically aggressive tumor phenotype with a systemic dissemination potential, rather than being a direct cause of disease progression (49–51). More recently, Faries (51) emphasized that SLN metastases serve primarily as markers of systemic disease rather than as effective barriers to metastatic dissemination, reinforcing the concept that extensive lymphadenectomy may not significantly impact the clinical outcomes (51).

In this context, the lack of prognostic benefit from extensive lymphadenectomy could be explained by the hypothesis that removal of additional nodes beyond the primary metastatic site may not influence the systemic disease progression if tumor dissemination has already occurred.

Therefore, the results of the present study suggest that an extensive lymphadenectomy including bilateral removal of the mandibular and retropharyngeal nodes may not improve outcome and therefore the increased surgical dose and potentially morbidity compared to ipsilateral regional/sentinel lymphadenectomy may not be justified by an oncological benefit. This is also supported by the higher incidence of metastasis found in this series of dogs at the level of the ipsilateral mandibular LNs. On the other hand, the extirpation of the ipsilateral mandibular LNs only may result in undetected nodal metastases.

To reduce the risk of missing nodal metastases–potentially compromising the oncological outcome–while still minimizing the surgical dose, SLN mapping techniques should be preferred over regional lymphadenectomy; these techniques indeed allow for removal of the nodes at highest risk of harboring nodal metastases while reducing the number of excised nodes compared to elective neck dissection (40, 43, 52).

In this study, CTL was performed only in 8 dogs. The SLNs were accurately identified in all but one case. As previously reported, CTL has a reported accuracy of up to 97% for oral tumors in dogs (63). The limited use of indirect CTL or other preoperative/intraoperative sentinel mapping techniques in the present study represents a significant limitation, thus preventing the authors from confirming the efficacy of CTL and assessing its impact on the choice between a radical vs. a more selective lymphadenectomy procedure.

Another important limitation of this study is the absence of a control group of dogs in which lymphadenectomy was not performed, impeding the evaluation of the real role of cervical lymphatic metastasis in prognosis. Nevertheless, it should be outlined that the non-removal of the regional (or better, sentinel) LNs does not represent the current “standard of care” for OMM; in fact, in this scenario, only the overt metastatic LNs would have been diagnosed and excised, thus underestimating the real clinical stage in case of micro metastatic LNs.

A further limitation of this study is the lack of a control group of dogs that underwent surgery and lymphadenectomy without adjunctive vaccine treatment. The choice to include only dogs with OMM treated by surgery and adjuvant anti-CSPG4 electrovaccination was to ensure a homogeneous population of patients for statistical analysis. Nevertheless, this may have introduced a confounding factor when assessing the impact of lymphadenectomy on MST and DFI. The anti-CSPG4 vaccine may have contributed to limiting the metastatic spread, thereby potentially influencing the correct evaluation of the prognostic significance of LN excision on outcome.

Additionally, the potential variability in the number of LNs removed by the surgeons involved in this study could have resulted in an underestimation of LN metastasis in some cases and, consequently, even of the definitive clinical stage. This was not the case for the medial retropharyngeal LN that, being usually a single LN, was never missed if the goal was to remove it, uni-or bilaterally. Nevertheless, missed LNs, if not enlarged, may have occurred intraoperatively for a lateral retropharyngeal LN, present in about 1/3 of dogs only, and the mandibular LNs, present in dogs in a variable number of 2–3 up to 5 on each side (53, 54). Missed LNs may account for the development of subsequent nodal metastases, even in dogs that underwent total bilateral lymphadenectomy or excision of nodes within the same lymph center in cases of mandibular lymphadenectomy. The prognostic impact of a missed LN is at present unknown. Strict monitoring and restaging during the follow-up may permit a quick detection and treatment of new LN metastases.

The results recorded in this study apply only for this specific cohort of dogs and a translation to canine OMMs differently treated requires further investigations. However, based on this study, it can be concluded that (1) a bilateral mandibular and medial retropharyngeal lymphadenectomy, while ensuring the highest detection rate of LN metastasis, was not associated with a significative survival improvement, and (2) an ipsilateral mandibular lymphadenectomy (at least, prudently, for an ipsilateral OMM, unless differently dictated by the preoperative clinical, cytological and imaging findings) may be a reasonable option as it was here associated with a high probability of removing the nodes characterized by the highest risk of metastasis. Nevertheless, apart from the excision of the clearly metastatic LNs already at presentation, authors emphasize the absolute need to implement the procedures aimed at identifying SLN or, ambitiously, those LNs apparently normal but microscopically metastatic. In all circumstances, after the initial wide surgical excision followed by the adjuvant treatment chosen among those the clinician feels more confident with, a strict monitoring and restaging during the follow-up may permit further therapeutic procedures with the ultimate goal of prolonging the survival.

## Data Availability

The raw data supporting the conclusions of this article will be made available by the authors, without undue reservation.

## Footnotes

^1^ www.graphpad.com

^2^ www.medcalc.org

## Appendix material

A power analysis based on the log-rank test was performed to calculate the number of dogs required in each group to conduct the statistical analyses. An alpha error of 0.05 and a statistical power of 80% were assumed. When the population was stratified according to the presence or absence of lymph node metastases at diagnosis, a 1-year survival rate of 50% was hypothesized based on Authors clinical experience for dogs with lymph node metastases (Group A) and 80% for those without lymph node metastases (Group B). According to Boston et al. (12) the number of patients in Group A was approximately half that of Group B.

Similarly, when the population was stratified based on the type of lymphadenectomy performed, a 1-year survival rate of 50% was hypothesized based on Authors clinical experience for dogs that underwent ipsilateral lymphadenectomy (Group 1), and 80% for those that underwent bilateral lymphadenectomy (Group 2). According to Camerino et al. (29) the number of dogs in Group 1 was approximately 1.3 times higher than in Group 2. We collected a population that mirrors the indications provided by the preliminary statistical analyses. It should be noted that subgroup comparisons were performed with the awareness that the statistical power may be reduced due to the limited number of cases.
